# External iliac arterial dissection after robot‐assisted radical cystectomy with an intracorporeal ileal conduit and extended pelvic lymph node dissection

**DOI:** 10.1002/iju5.12650

**Published:** 2023-10-04

**Authors:** Syunsuke Nakashima, Shinro Hata, Mayuka Shinohara, Tadasuke Ando, Toshitaka Shin, Hiromitsu Mimata

**Affiliations:** ^1^ Department of Urology Oita University Faculty of Medicine Yufu Oita Japan

**Keywords:** arterial dissection, complication, extended pelvic lymphadenectomy, percutaneous thrombectomy, robotic surgery, stent placement

## Abstract

**Introduction:**

External iliac arterial dissection after robot‐assisted laparoscopic procedures is an extremely rare complication. It may cause severe adverse outcomes, such as lower limb necrosis.

**Case presentation:**

A 73‐year‐old man was diagnosed with cT2 ≤ N0M0 bladder cancer and underwent robot‐assisted radical cystectomy. After surgery, he complained of severe left lower leg pain. Computed tomography showed external iliac arterial occlusion. Furthermore, the emergency operation revealed external iliac arterial dissection and occlusion.

**Conclusion:**

The robot arm does not have any sense of force or touch. Thus, it is necessary to pay sufficient attention to the traction of blood vessels and contact with major organs.

**Abbreviations & Acronyms**
CT: computed tomography
DVT: deep vein thrombosis
RARC: robot‐assisted radical cystectomy

**Keynote message**

External iliac artery dissection is a rare and serious complication in robotic surgery, but early treatment can prevent lower extremity ischemia.

## Introduction

Robot‐assisted laparoscopic surgery has been performed in various fields, not only in urology. As a major advantage, it allows the performance of delicate procedures using robotic arms. However, because the surgeon has no sense of touch, he/she may unknowingly cause damage to surrounding organs. Moreover, external iliac arterial dissection after robot‐assisted laparoscopic procedures is an extremely rare complication. It often results in critical limb ischemia, requiring emergency surgery. To the best of our knowledge, no study has reported external iliac arterial dissection after robot‐assisted laparoscopic surgery. Here, we describe a case of external iliac arterial dissection after RARC with an intracorporeal ileal conduit urinary diversion and extended pelvic lymphadenectomy.

## Case presentation

**Fig. 1:**
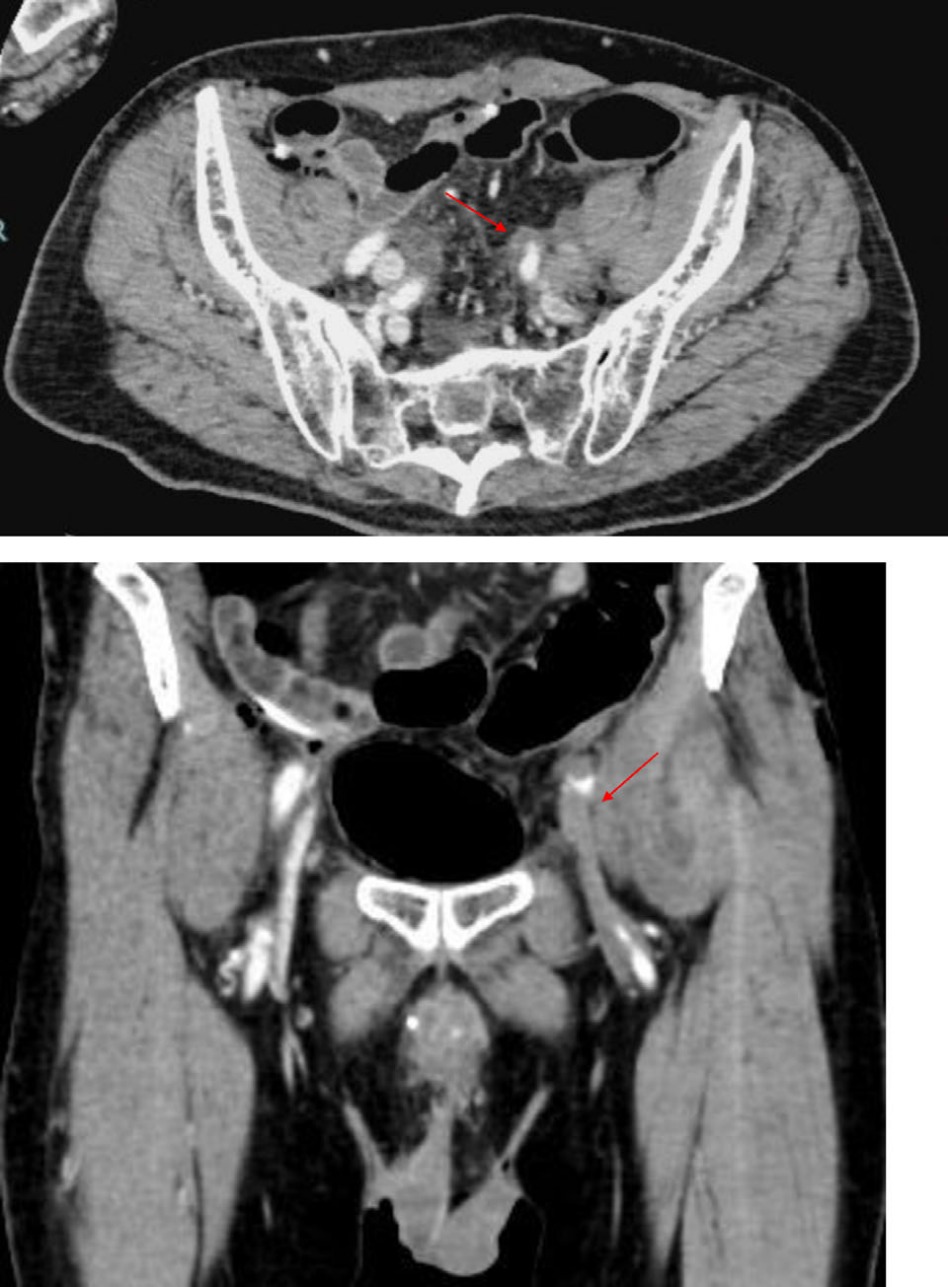
Arrow in CT image shows an external iliac arterial dissection and thrombotic occlusion.

**Fig. 2:**
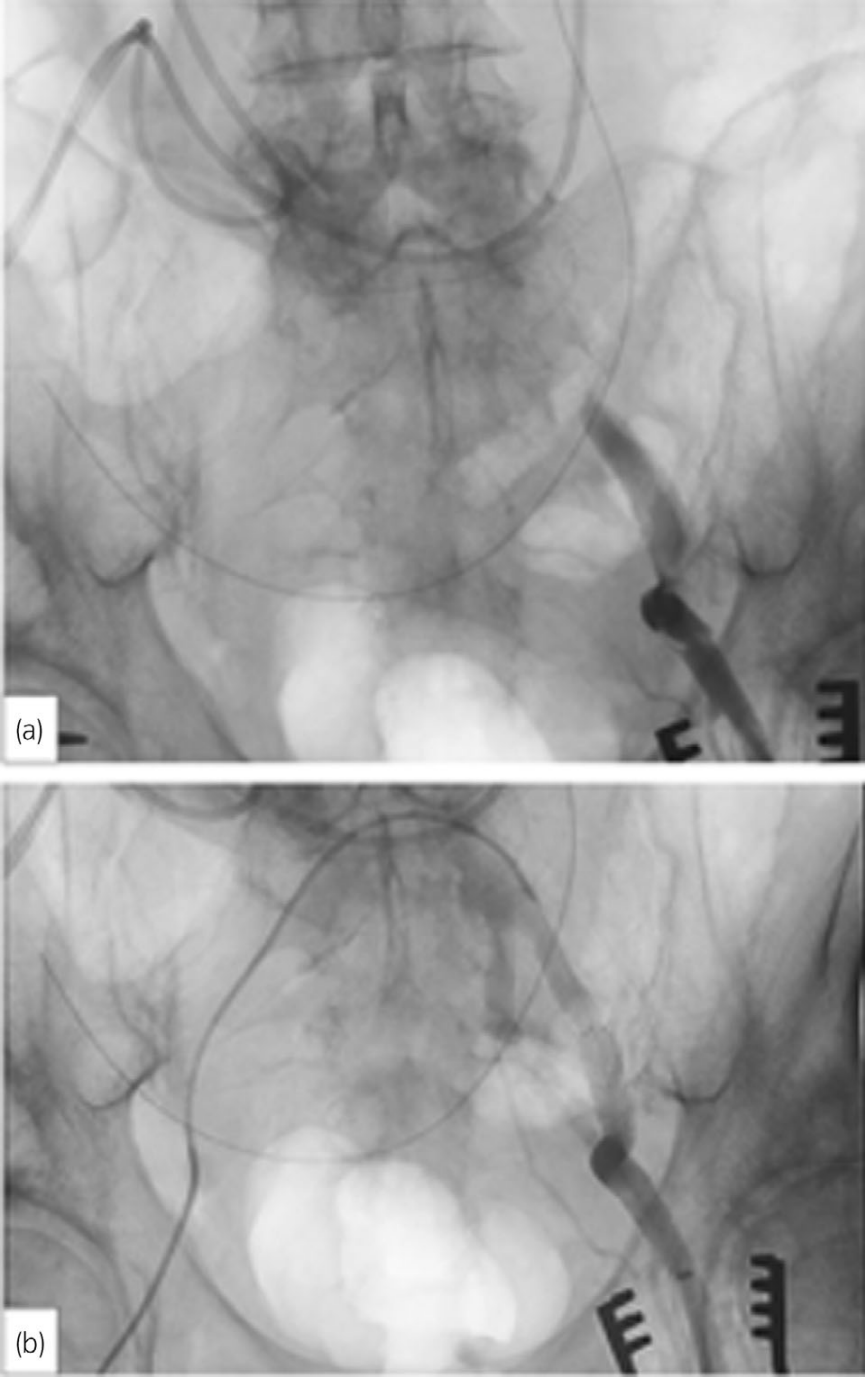
Angiography findings. (a) The angiography shows an external iliac arterial occlusion before stent placement. (b) After stent placement, an external iliac artery has good blood flow.

A 73‐year‐old man was admitted to our hospital for macroscopic hematuria. Cystoscopy revealed a nodular tumor filling the right wall of the bladder. He underwent transurethral resection of the bladder cancer and was diagnosed with urothelial carcinoma, high grade, and pT2≤. CT did not detect obvious lymph node swelling and distant metastasis. Moreover, he underwent RARC with an intracorporeal ileal conduit urinary diversion and extended pelvic lymphadenectomy after three cycles of neoadjuvant chemotherapy with gemcitabine and cisplatin. The total operative and console times were 754 and 546 min, respectively. The amount of blood loss was approximately 225 mL. After returning to the hospital room from the operating room, he complained of severe pain in the left lower leg. Pulseless of the dorsal pedal artery, pallor, paresthesia, and motor paralysis were not observed. We suspected compartment syndrome owing to the prolonged lithotomy position and consulted an orthopedist. He was diagnosed with compartment syndrome and underwent an emergency fasciotomy of the left lower leg. However, the skin coloration and pain in his left lower leg did not improve. Pulseless of the dorsal pedal artery and pallor were observed the next day. Paresthesia and motor paralysis were not observed. CT revealed an external iliac arterial dissection and thrombotic occlusion (Fig. 1). We consulted a cardiovascular surgeon. He underwent an emergency percutaneous thrombectomy and stent placement (Fig. 2). After this surgery, he started to take aspirin. Consequently, his left lower leg pain improved and he was discharged 46 days after RARC. His clinical course was uneventful through follow‐up.

## Discussion

External iliac arterial dissection is a rare complication of RARC with an intracorporeal ileal conduit urinary diversion and extended pelvic lymphadenectomy. To the best of our knowledge, no similar previous case has been reported. The major vascular complications of robotic surgery are DVT and compartment syndrome. However, their frequencies are very low, with 0.5–0.6% and 0.03–0.3%, respectively.^1^ Vascular injuries most frequently occur during lymphadenectomy and trocar insertion.^2^ Lymphadenectomy is associated with the highest risk for retroperitoneal injuries of large (aorta, vena cava, and iliac) vessels.^3^

When a blunt external force is applied to an artery, damage occurs to the less elastic intima, causing arterial dissection.^4^ In addition, hematoma formation leads to the blockage of the vessel lumen. In this case, traction was required during pelvic lymph node dissection because the external iliac artery was tortuous and covered the lymph nodes. Thus, the traction time for the lymph node dissection was extended. In this case, the mechanism of injury is presumed to have been excessive traction and direct contact. If the artery is compressed or pulled during the dissection, we should pay attention to carefully observe the arterial pulsation and palpate the femoral artery pulsation from the body surface after the dissection is completed.

Vascular occlusion caused by traumatic iliac artery deviation differs from chronic diseases, such as atherosclerosis obliterans due to poorly developed collateral blood vessels. Therefore, ischemic symptoms in the lower limbs are progressive, and delay in diagnosis and treatment can cause lower limb necrosis, making it difficult to save the limbs.^5^

Asymptomatic cases are treated conservatively. However, in the case of severe symptoms of lower limb ischemia due to arterial occlusion, it is necessary to take the dynamic CT as soon as possible.^5^ The rate of limb amputation due to lower extremity arterial occlusion is 5–30%. The golden time for treatment is 6–8 h. In this case, the patient had persistent pain in the lower limb even on the day after surgery. Thus, emergency surgery for lower limb ischemia was immediately performed, and the patient achieved positive outcomes.

We assumed that the patient had compartment syndrome since he had lower limb pain after prolonged surgery in the head‐down lithotripsy position. If findings suggest arterial occlusion, contrast‐enhanced CT should be immediately performed after the visual examination of the entire lower limb and palpation of each artery, not just the lower leg. If a diagnosis of serious vascular occlusion due to arterial deviation is made, as in this case, prompt surgery is essential.

The robot arm does not have any sense of force or touch. Therefore, it is necessary to pay sufficient attention to the traction of blood vessels and contact with major organs.

## Conclusion

We presented our experience of a rare case of external iliac arterial dissection after RARC. External iliac arterial dissection after RARC is an extremely rare complication, however, may lead to serious adverse outcomes, such as lower limb necrosis. Thus, the traction of blood vessels and contact with major organs by the robot arms should be avoided as much as possible.

## Author contributions

Syunsuke Nakashima: Writing – original draft. Shinro Hata: Writing – review and editing. Mayuka Shinohara: Data curation. Tadasuke Ando: Visualization.Toshitaka Shin: Writing – review and editing. Hiromitsu Mimata: Conceptualization.

## Conflict of interest

The authors declare no conflict of interest.

## Approval of the research protocol by an Institutional Reviewer Board

Not applicable.

## Informed consent

Not applicable.

## Registry and the Registration No. of the study/trial

Not applicable.
